# Oligodendrocytes matter: a review of animal studies on early adversity

**DOI:** 10.1007/s00702-023-02643-4

**Published:** 2023-05-03

**Authors:** Mate Abraham, Jutta Peterburs, Annakarina Mundorf

**Affiliations:** 1 Division of Experimental and Molecular Psychiatry, Department of Psychiatry, Psychotherapy and Preventive Medicine, LWL University Hospital, Ruhr-University Bochum, Bochum, Germany; 2 Institute for Systems Medicine and Department of Human Medicine, MSH Medical School Hamburg, Hamburg, Germany

**Keywords:** Psychiatry, Neurodevelopmental, White matter, Myelinization, Neuronal maturation, Hemispheric asymmetry

## Abstract

Exposure to adversities in early life appears to affect the development of white matter, especially oligodendrocytes. Furthermore, altered myelination is present in regions subjected to maturation during the developmental time when early adversities are experienced. In this review, studies applying two well-established animal models of early life adversity, namely maternal separation and maternal immune activation, focusing on oligodendrocyte alterations and resulting implications for psychiatric disorders are discussed. Studies revealed that myelination is reduced as a result of altered oligodendrocyte expression. Furthermore, early adversity is associated with increased cell death, a simpler morphology, and inhibited oligodendrocyte maturation. However, these effects seem to be region- specific as some brain regions show increased expression while others show decreased expression of oligodendroglia-related genes, and they occur especially in regions of ongoing development. Some studies furthermore suggest that early adversity leads to premature differentiation of oligodendrocytes. Importantly, especially early exposure results in stronger oligodendrocyte-related impairments. However, resulting alterations are not restricted to exposure during the early pre- and postnatal days as social isolation after weaning leads to fewer internodes and branches and shorter processes of oligodendrocytes in adulthood. Eventually, the found alterations may lead to dysfunction and long-lasting alterations in structural brain development associated with psychiatric disorders. To date, only few preclinical studies have focused on the effects of early adversity on oligodendrocytes. More studies including several developmental stages are needed to further disentangle the role of oligodendrocytes in the development of psychiatric disorders.

## Introduction

Emerging evidence points toward alterations in oligodendrocytes and myelination leading to impairments associated with psychiatric disorders such as schizophrenia and depression (Schmitt et al. 2019; Abraham et al. 2022). Translational studies further point to replicable alterations in white matter integrity visible in imaging and neuropathological studies in subjects suffering from depression across multiple species (Abraham et al. 2022). Interestingly, here, a particular role of early life adversities in the development of white matter alterations in depression is highlighted. Findings indicate that stress interferes with physiological myelination patterns. Furthermore, altered myelination is present in regions subjected to maturation during the developmental time when early adversities are experienced (Abraham et al. 2022). Another interesting finding is reduced density of oligodendrocytes in the gyral white matter of depressive patients that experienced childhood abuse (Lutz et al. 2017; Tanti et al. 2018). Similar results have been reported for patients diagnosed with schizophrenia (Fessel 2022; Schmitt et al. 2019). Moreover, recent findings propose a shift towards a more mature oligodendrocyte phenotype in patients exposed to childhood abuse, an alteration which is hypothesized to be driven by an increase in the rate of differentiating oligodendrocytes (Tanti et al. 2018; Abraham et al. 2022).

Stereological post-mortem studies on oligodendrocytes and myelination that investigated number and density as well as spatial distribution of oligodendrocytes via Nissl-staining consistently report decreased numbers of oligodendrocytes in patients diagnosed with schizophrenia. More precisely, a decrease in the numbers of cortical layer III oligodendrocytes as well as in the white matter of almost 30% has been found with immunostaining (Hof et al. 2003). Moreover, when studying numbers and densities of neurons, oligodendrocytes and astrocytes in the posterior hippocampal subregions, a significant decrease in the mean number of oligodendrocytes was found in the left and right CA4 in patients compared to controls (Schmitt et al. 2009). A similar study focusing on the anterior part of the hippocampus revealed fewer oligodendrocytes in the left CA4 (Falkai et al. 2016). This result was also confirmed in an independent replication study (Schmitt et al. 2022). In affective disorders, stereological studies have not yet yielded consistent results. Some studies reported reduced oligodendrocyte density in patients diagnosed with depression (Lutz et al. 2017; Tanti et al. 2018), but others found the opposite. For example, a stereological post-mortem study including patients diagnosed with major depression, patients with bipolar disorder, and controls found different alterations in the posterior hippocampus depending on the disorder. In brief, patients with major depression demonstrated a higher density of oligodendrocytes in CA2/3, CA4 and the subiculum whereas patients with bipolar disorder had more oligodendrocytes solely in CA1 when compared to controls (Malchow et al. 2015).

From a translational perspective, alterations in oligodendrocyte maturation are present across humans and mice. For example, mice exposed to chronic stress show longer and thicker processes of mature oligodendrocytes with a higher density, while the density and morphology of oligodendrocyte progenitor cell processes was unaltered. Interestingly, studies have reported that neither microglia activation, nor the number of astrocytes was affected by chronic stress (Miyata et al. 2016), implying that mature oligodendrocytes are more strongly affected by stress exposure than other neuroglia cells (Miyata et al. 2016).

In general, oligodendrocytes are a type of neuroglia cells generated from oligodendrocyte progenitor cells (Jakovcevski et al. 2009; Kuhn et al. 2019). They provide an electric insulation for axons of the central nervous system by creating the myelin sheath. Therewith, they enable saltatory conduction of action potentials and proper neuronal function (Jakovcevski et al. 2009). Given that the myelinization of axons is an energy-consuming process associated with a high metabolic turnover, oligodendrocytes are especially vulnerable to cytotoxic and excitotoxic factors (Kuhn et al. 2019). A detailed explanation of oligodendrocyte development and function is beyond the scope of this review. The interested reader is referred to the excellent review by Kuhn et al. (2019). Given that brain development is an ongoing process until adulthood and that several brain regions undergo rapid development during particular periods, disruption in early brain development may render the brain vulnerable to the onset of psychopathologies (Lupien et al. 2009; Meyer and Lee 2019). A disbalance in oligodendrocyte maturation or oligodendrocyte progenitor cells especially in early life may be key to disentangling neuronal alterations found in psychiatric patients.

In rodents, a well-established and widely used model to induce early life stress is the maternal separation (MS) paradigm (Lumertz et al. 2022; Waters and Gould 2022). Here, the pups are separated from the dam for several hours a day for a defined period (mostly the first two–three weeks after birth), resulting in neurobiological and behavioral impairments in pups (Mundorf et al. 2020; Lumertz et al. 2022; Waters and Gould 2022) as well as in dams (Bölükbas et al. 2020; Mundorf et al. 2022). Another frequently used animal model for early life stress and neurodevelopmental disorders, especially for influencing prenatal brain development, is the maternal immune activation (MIA) model (Haddad et al. 2020; Białoń and Wąsik 2022). In this model, the effects of prenatal exposure to maternal inflammation are induced through the administration of a viral mimetic, e.g., polyinosinic-polycytidylic acid (Poly(I:C)), which leads to long-lasting impairments in the offspring (e.g., Juckel et al. 2021; Mueller et al. 2021; Mundorf et al. 2021a; Wegrzyn et al. 2021). MS and MIA are often used as models to study the development and effects of depression and schizophrenia, respectively (Vetulani 2013; Białoń and Wąsik 2022).

In general, these animal models can help to disentangle the role of stress-induced alterations of oligodendrocytes on brain development during gestation or in the early postnatal phase, when several brain regions are still maturing (Lupien et al. 2009). In a study conducted on pregnant female rhesus macaques, a total of thirteen animals were exposed to MIA either during the first or second trimester of pregnancy or were saline-injected (controls) (Page et al. 2021). At the age of 3.5–4 years, animals were sacrificed, and neuronal gene expression was analyzed for specific regions. Interestingly, conducting a cell-type enrichment analysis revealed that, among others, markers for oligodendrocyte precursor cells, but not markers for oligodendrocytes, were enriched among downregulated genes. In terms of regions, the hippocampus showed the most differentially expressed genes. For example, upregulated genes were strongly enriched in oligodendrocytes, whereas downregulated genes were strongly enriched in oligodendrocyte progenitor cells. Furthermore, co-expression network analysis identified region-specific alterations in synaptic signaling and oligodendrocytes. The authors concluded that one of the strongest effects of MIA is the altered transcription of genes related to oligodendrocytes and myelination, with a shift in the regulation of gene expression to genes expressed by more mature oligodendrocytes (Page et al. 2021). The results implying a shift towards a higher cellular activity in more mature oligodendrocytes offer a correlate to immunohistochemistry findings that identify a more mature oligodendrocyte phenotype after early life stress (Page et al. 2021).

Studies have suggested a driving effect of oligodendrocyte alterations after early life adversity for the development of disorders such as depression and schizophrenia (Abraham et al. 2022; Fessel 2022; Lutz et al. 2017; Tanti et al. 2018; Schmitt et al. 2019). However, in which form oligodendrocytes are altered after early life stress is still unclear. Thus, the present review aims to summarize what we know regarding the implications of early life adversity on oligodendrocytes from animal models, and to disentangle potential consequences on brain development. To this end, the scientific database PubMed was searched for articles published until November 2022 on oligodendrocytes and early life adversity in animal models (such as, but not limited to, MS or MIA). Studies focusing on undernutrition were excluded. This resulted in 10 studies included in this review.

## Early life adversity and oligodendrocytes in animal models

**Fig. 1:**
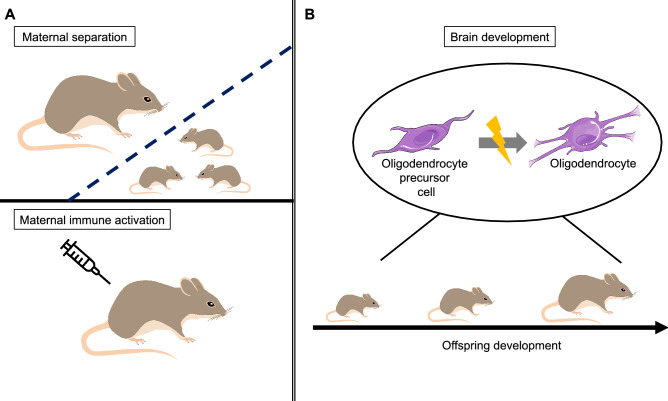
Different animal models of early adversity (**A**) lead to alterations in oligodendrocytes in the developing offspring (**B**). Early stress seems to affect oligodendrocyte maturation and alters the normal proportion of oligodendrocyte precursor cells and oligodendrocytes during development. Stress may also result in less complex oligodendrocyte morphology. The Figure was partly generated using Servier Medical Art, provided by Servier, licensed under a Creative Commons Attribution 3.0 unported license, the rat image is modified from Mostafa Elturkey from Pixabay

Animal models of early life adversity help to disentangle the role of stress-induced alterations of oligodendrocytes on brain development during gestation or in the early postnatal phase (Lupien et al. 2009). Specifically, the MIA model can elucidate potential effects of prenatal exposure, while the MS model can help to unravel the consequences of postnatal exposure. In the following, consequences of exposure on oligodendrocyte development and formation are summarized, first based on studies using the MIA model, followed by studies using the MS model of early adversity (see Table 1 and Fig. 1 for an overview).

**Table 1:** Animal models of maternal immune activation (MIA) and maternal separation (MS). MIA was performed by injecting Poly(I:C) at different gestational days (GD), MS was induced by separating pups from the dam at different postnatal days (PD). Additionally, the developmental age of analysis and neuronal alterations in oligodendrocytes (OL) are listed

Author, year	Strain	Animal model	Age analyzed	Oligodendrocytes
Makinodan et al. (2008)	C57BL/6 mice	MIA; GD 9.5	Early postnatal, juvenile, adult	Retarded myelination & axonal abnormalities in early postnatal stages, ↔ OL
Li et al. (2010)	C57BL6/N mice	MIA; GD9 or 17	Adults	↑ Fractional anisotropy ↓ CNPase-positive cells
Zhang et al. (2020)	Male C57BL6/N mice	MIA; GD9	Adults	↓ Expression of myelin- and OL-related genes
Bordner et al. (2011)	C57Bl/6 J (B6) x DBA/2 J (D2) mice	MS (PD2-5: 4 h/day + PD6-16: 8 h/day) + early weaning at PD17	PD17 (juveniles)	↓ Mature OL and terminal differentiation markers
Teissier et al. (2020)	BALB/cJ Rj mice	MS (PD2-14, 3 h/day)	PD15 (juveniles)	Premature OL differentiation
Wang et al. (2021)	C57BL/6 mice	MS from parents (PD2-12, 8 h/day); father-staying control	PD23 (weaning)	Altered oligodendroglial lineage cells ↓ Hippocampal oligodendrocyte precursor cells
Zhang et al. (2002)	Sprague Dawley x Long Evans rats	MS over 24 h	PD6, 12, 20	PD 6 + 12: ↑ cell death in regions of postnatal development + white matter
Yang et al. (2017)	Sprague Dawley rats	MS (PD3-21, 3 h/day)	Adults	↑ Oligodendrocyte precursor cells ↓ Mature oligodendrocytes
Zeng et al. (2020)	Sprague Dawley rats	MS (PD2-5: 4 h/day, PD6-16: 6 h/day)	Adults	Disrupted neuron-glia integrity ↓ Fractional anisotropy ↓ Oligodendrocyte maturation
Makinodan et al. (2012)	Male mice (not further specified)	Social isolation from PD21	Adults	↔ OL density ↓ OL internodes ↓ OL branches ↓ OL processes

### Maternal immune activation model of early life adversity

Makinodan et al. (2008) investigated myelination and axonal development in the hippocampus of MIA offspring. In this study, pregnant C57BL/6 mice were injected at gestational day 9.5 with either Poly(I:C) or saline (controls) and the offspring was tested. Histological staining of early postnatal, juvenile, and adult mice brains indicates that MIA led to retarded myelination and axonal abnormalities in early postnatal stages but showed normalization until adulthood. However, even though myelination was reduced in the early postnatal phase, the number of oligodendrocytes did not differ between groups.

Li et al. (2010) used voxel-based analysis of postnatal white matter microstructure in the MIA mouse model. Here, pregnant C57BL6/N mice were intraperitoneally injected with Poly(I:C) in early (gestational day 9) or late pregnancy (gestational day 17) and the offspring was scanned at 12-week-old using diffusion tensor imaging. In the early exposure group, changes in fractional anisotropy were more extensive compared to controls. This was true for regions with increased and decreased fractional anisotropy. The late exposure group also showed several regions with increased fractional anisotropy compared to controls, but less extensive than the early exposure group. Of note, the observed fractional anisotropy anomalies were predominantly left-sided. To further quantify the number of oligodendrocytes via immunohistochemistry, the marker 2′,3′-cyclic nucleotide 3′-phosphodiesterase (CNPase) was used. Interestingly, regions showing a decreased or an increased number of CNPase-positive cells corresponded with the tracts showing decreased or increased fractional anisotropy, respectively. This finding further supports the notion that fractional anisotropy is a suitable imaging marker to determine oligodendrocyte density without the necessity to sacrifice animals, thus allowing for repeated testing in the same individual.

Zhang et al. (2020) analyzed neuronal expression of oligodendroglia-related genes in adult male C57BL6/N mice that were prenatally exposed to Poly(I:C). Pregnant dams were injected with Poly-I:C via the tail vein on gestation day 9, and the myelin- or oligodendroglia-related genes *SOX-10*, *MAG*, and *Tf* were analyzed in adult offspring by in situ hybridization and quantitative real-time PCR. In situ hybridization revealed that expression of *SOX-10*, *MAG*, and *Tf* was reduced in the bilateral limbic system including the hippocampus, retrosplenial cortex, and parahippocampal gyrus in MIA-exposed mice. Similar results were obtained by quantitative real-time PCR, with reduced expression levels of *SOX10, MAG*, and *Tf* in the bilateral medial prefrontal cortex, sensory cortex, amygdala, and hippocampus in the MIA group. The authors hypothesized that MIA during pregnancy could lead to compromised white matter connectivity as seen in neurodevelopmental disorders such as autism spectrum disorders.

In sum, prenatal MIA seems to reduce myelination by altering oligodendrocyte expression patterns. However, these changes seem to be region-specific, as some brain regions show increased expression while others show decreased expression of oligodendroglia-related genes. Moreover, these alterations could change through development. To date, only few studies focus on the effects of MIA on oligodendrocytes. More studies spanning several developmental stages are needed to further disentangle the effect of MIA on oligodendrocytes.

### Maternal separation model of early life adversity

Bordner et al., (2011) applied a combined approach of MS together with early weaning in mice followed by extensive molecular analyses of the medial prefrontal cortex. Pups from a C57Bl/6 J (B6) and DBA/2 J (D2) breed were either left undisturbed and weaned at postnatal day 23 (controls) or separated from the dam for 4 h per day from postnatal days 2–5, and for 8 h per day on postnatal days 6–16, followed by weaning on postnatal day 17. Compared to controls, the separated pups showed downregulation of markers of mature oligodendrocytes as well as of markers associated with processes such as the terminal differentiation of oligodendrocyte progenitor cells into myelinating oligodendrocyte progenitor cells. This downregulation was present in RNA, protein, and DNA methylation levels.

Teissier et al. (2020) exposed BALB/cJ Rj mice to MS to examine the effects of MS on oligodendrogenesis. Here, pups were separated for 3 h daily starting at postnatal day two until day 14. Results indicated that MS leads to premature oligodendrocyte differentiation at postnatal day 15 in the medial prefrontal cortex which results in the depletion of the oligodendrocyte progenitor pool in adulthood.

Wang et al. (2021) employed a slightly different MS protocol where pups are separated from both parents and the control pups are separated from their mother but stay with their father during the MS period. In detail, C57BL/6 wild-type mice were isolated for 8 h daily from postnatal day 2–12 from both parents (MS group) or only from the mother (controls). From postnatal day 12–23, all pups stayed with their parents. Neurobiological analyses at postnatal day 23 demonstrated that MS resulted in altered oligodendroglial lineage cells in the hippocampus and cortex as well as in a reduction of hippocampal oligodendrocyte precursor cells. Interestingly, when chronologically tracing the alterations in oligodendrocyte precursor cells, it was shown that the reduction was already present at postnatal day four.

Zhang et al. (2002) tested the effect of a one-time separation on cell death in the offspring of Sprague Dawley female rats and Long Evans male rats. Therefore, half of the pups per litter were separated from their mother and siblings over 24 h and sacrificed right after. The rate of neuronal cell death was measured in separated and non-separated pups with a variant of TUNEL staining by labeling the 3′ end of DNA fragments using terminal transferase (ApopTag). To investigate the developmental effects of separation, pups were separated for 24 h on postnatal days 6, 12, or 20. Interestingly, increased cell death rates were present in regions where postnatal development is prominent such as the cerebral and cerebellar cortex, and in several white matter tracts, but not in subcortical areas such as the thalamus and hypothalamus. The reported increased cell death was furthermore more pronounced in pups separated at postnatal days 6 and 12 but not in pups separated at postnatal day 20. The authors hypothesized that the increased cell death identified in white matter tracts could be a result of oligodendrocyte cell death, as these represent the predominant cell type in white matter.

Yang et al. (2017) analyzed medial prefrontal cortex myelination in Sprague Dawley rats with MS conducted from postnatal day 3–21 for 3 h daily. The researchers found long-lasting alterations until adulthood such as increased numbers of oligodendrocyte precursor cells and decreased numbers of mature oligodendrocytes after MS.

Zeng et al. (2020) studied neuron‐glia integrity with non‐invasive neuroimaging methods in adult Sprague–Dawley rats subjected to MS. The MS paradigm comprised separation periods of 4 h on postnatal days 2–5 and 6 h on postnatal days 6–16, and rats were scanned on postnatal days 60–62. Results revealed that MS led to disrupted neuron-glia integrity in the prefrontal cortex and the dorsal hippocampus, as indicated by changes in neurotransmitter levels such as glutamate, GABA, and N-acetyl-aspartate, as well as a globally found decrease in fractional anisotropy. Moreover, analyzing neurotransmitter levels via western blotting in the prefrontal cortex demonstrated inhibited oligodendrocyte maturation and myelination after MS.

In a study that examined the effects of social isolation, Makinodan et al. (2012) investigated the consequences of stress exposure after weaning on oligodendrocyte maturation and myelination. To this end, male mice expressing the enhanced green fluorescent protein in oligodendrocytes under the control of the proteolipid protein (PLP) promoter (PLP-eGFP) were housed upon weaning (postnatal day 21) either in isolation, in groups of four, or in an enriched environment (meaning larger cages with eight mice and novel toys every two days). At postnatal day 65, the medial prefrontal cortex was analyzed in terms of myelination and oligodendrocytes. When comparing all three groups, no difference in oligodendrocyte density was found and oligodendrocyte morphology did not differ between group-housed or environmentally enriched housed mice. But oligodendrocytes from socially isolated mice had a remarkably simpler morphology, with fewer internodes and branches and shorter processes. Additional electron microscopy revealed that the myelin thickness was reduced while the myelinated axon diameter was unaffected in isolated mice. Results also revealed that the critical window when social isolation altered oligodendrocyte morphology was between postnatal day 21 and 35, and that environmentally enriched housing of mice exposed to social isolation did not normalize the simpler morphologies of oligodendrocytes.

To summarize, the MS model of postnatal stress exposure seems to be associated with increased cell death as well as a simpler morphology of oligodendrocytes, along with inhibited oligodendrocyte maturation. Some studies point towards premature oligodendrocyte differentiation resulting from MS. So far, it is not clear whether MS results in a decrease or an increase of oligodendrocyte precursor cells. The reviewed studies point to a crucial role of the timepoint of exposure and developmental age of analysis, which could affect the localization of alterations, with regions of ongoing development being especially affected.

## Discussion

Early life adversities seem to affect the development of white matter by altering oligodendrocyte differentiation and maturation. Studies applying the animal models MIA and MS revealed that myelination is reduced consequently to altered oligodendrocyte expression. Furthermore, early adversity is associated with increased cell death, a simpler morphology as well as inhibited oligodendrocyte maturation. These alterations seem to be region-specific and occur especially in regions of ongoing development. In this context, oxidative stress represents a possible link between external stressors and cellular alterations. Oxidative stress has been shown to be more strongly present in post-mortem probes of individuals having suffered from depression (Szebeni et al. 2017; Abraham et al. 2022). Oxidative stress causes multiple cellular alterations, among which are damage to cell organelles and DNA alterations. Reparation of these alterations is an energy-consuming process, thus bearing the potential to deplete cellular energy reserves. In severe cases, DNA alterations can even lead to apoptosis, possibly resulting in alterations in myelination and therewith in fractional anisotropy. Aberrations in myelination could further be driven by the fact that repairing cellular damage is an extraordinarily energy-consuming process, which could in turn lead to decreased myelin production, as this represents a very energy-intensive process itself (Szebeni et al. 2017).

Some studies furthermore suggest that early adversity leads to premature differentiation of oligodendrocytes. Importantly, time of exposure seems to be critical for determining the impact on oligodendrocytes, with especially early exposure resulting in stronger oligodendrocyte-related impairments (Zhang et al. 2002; Li et al. 2010; Makinodan et al. 2012). Interestingly, resulting effects are not restricted to exposure during the early pre- and postnatal days, given that social isolation after weaning leads to fewer internodes and branches and shorter processes of oligodendrocytes in adulthood (Makinodan et al. 2012).

The different windows of susceptibility to oligodendrocyte alterations may be related to the developmental periods of brain maturation (Lupien et al. 2009). The reviewed studies highlight that the observed region-specific impairments in oligodendrocytes are linked to altered myelination in regions subjected to maturation during the developmental time when early adversities are experienced. Lupien and colleagues (2009) impressively reviewed how the consequences of early adversity at different stages in life depend on the brain areas that are developing at the time of exposure highlighting specific periods when regions are most sensitive to increased stress hormone exposure, as well as periods when several regions experience accelerated growth. Given that for example the amygdala, the frontal cortex, and the hippocampus experience a prolonged development, these regions are thus most sensitive to stress exposure.

Considering that growth as well as formation of important neuronal networks and circuitries is linked to myelinization (Tau and Peterson 2010; Almeida and Lyons 2017), abnormalities in oligodendrocyte maturation or differentiation may play a significant role in the development of cognitive or emotional alterations such as increased anxiety or reduced memory performance. The reported earlier maturation of oligodendrocytes (Teissier et al. 2020) is in line with accelerated brain maturation as a consequence of early adversity observed in several species (Callaghan and Tottenham 2016; Chaudhari et al. 2022). Earlier maturation of oligodendrocytes along with simpler morphology and reduced differentiation may lead to dysfunction and long-lasting alterations in structural brain development as found in humans after early deprivation in childhood due to institutionalization (Kumsta et al. 2017; Sheridan et al. 2022). However, further studies across different species are needed to determine the exact role of oligodendrocyte alterations in the context of depressive and schizophrenia-like phenotypes.

Another interesting point are hemispheric differences in impaired oligodendrocyte development. For instance, Li and colleagues (2010) reported predominantly left-sided alterations. Others found reduced oligodendrocyte-related gene expression in both hemispheres, e.g., Zhang et al (2020). Unfortunately, most studies did not report hemisphere-specific alterations and thus, it is unclear whether both hemispheres were equally affected or whether results were simply not analyzed in a hemisphere-specific manner. Nonetheless, hemispheric asymmetries or symmetries in oligodendrocyte alterations are an important avenue for future research. Indeed, hemispheric asymmetries in white matter structures are well known (Honnedevasthana Arun et al. 2021; Ocklenburg and Mundorf 2022) and have been observed in the context of psychiatric disorders such as depression and schizophrenia (Ho et al. 2017; Mundorf and Ocklenburg 2021; Mundorf et al. 2021b) as well as in preclinical rodent models of psychiatric disorders (Mundorf and Ocklenburg 2023). Along these lines, it would be relevant to investigate in future animal studies whether the reported alterations in oligodendrocyte maturation show hemispheric asymmetries, or whether existing hemispheric asymmetries are reduced by early life adversity.

Important to note are temporal and spatial differences between humans and rodents. Humans have an especially prolonged brain maturation and myelination process compared to rodents. While forebrain myelination only requires several weeks in mice and rats, it takes years in humans (Jakovcevski et al. 2009). Prolonged brain development in humans is largely due to numerous neocortical regions being present that are lacking in rodents. On the other hand, certain phylogenetically old brain structures, such as the olfactory bulb, are underdeveloped in humans compared to rodents, thus further impeding translational comparison (Bayer et al. 1993; Jakovcevski et al. 2009). Prolonged brain development is paralleled by differences in cellular composition during brain development between humans and rodents: while markers of early oligodendrocyte progenitor cells, such as PGFRα, can be detected in the mouse at embryonic day 15 and reach a peak at postnatal day 14, the same markers could only be detected in humans in the 10th gestational week, with ongoing myelination detectable years after birth (Bayer et al. 1993; Jakovcevski et al. 2009).

On the genetic level, several hypotheses have been put forward concerning the impact of oligodendrocyte-related genes in animal models. One recent model that has gained considerable attention is the Tcf4/Olig2 model. Tcf4 and Olig2 are transcription factors that seem to play a crucial role in oligodendrocyte differentiation and myelination. More precisely, a heterodimerization of these proteins appears to be crucial in controlling oligodendrocyte differentiation and myelination (Wedel et al. 2020). Deleting the long isoform of Tcf4 has been shown to lead to substantially impaired terminal oligodendrocyte differentiation in mice (Wedel et al. 2020). Moreover, impairment of Tcf4-expression in humans has been associated with psychiatric illnesses, such as schizophrenia and autism spectrum disorders (Wedel et al. 2020).

In addition, environmental factors have been shown to effect numerous genes associated with oligodendrocyte differentiation and myelination. Early life stress has been demonstrated to lead to alterations in the expression of genes related to myelination in rodents (Gutman and Nemeroff 2002; Tractenberg et al. 2016; Teissier et al. 2020). Furthermore, hypomyelination following early life stress seems to represent a robust translational finding. Rodents subjected to early life stress have been repeatedly shown to develop hypomyelination (Gutman and Nemeroff 2002; Makinodan et al. 2008; Tractenberg et al. 2016). In humans, experience of early life adversities has been shown to lead to hypomyelination in phylogenetically new areas executing complex regulatory functions, such as the medial prefrontal cortex (Insana et al. 2016; Lutz et al. 2017; Nelson 2017).

Taken together, the overall conclusion of the present review is limited by the small number of included studies as well as several differences in research design and methodology. However, most studies do point towards substantial alterations of oligodendrocytes due to early adversity. In light of a translational perspective, it is important to note that between rodents and humans, relevant differences in temporal and spatial distribution and regulatory signals for oligodendrocyte differentiation are present that may influence the exceptional susceptibility of humans to demyelinating diseases (Jakovcevski et al. 2009).

Comparisons between human and rodent forebrains have delineated that more studies are urgently needed to eventually unravel the implications on oligodendrocytes and their role in the development of psychiatric disorders after early exposure.

## Data Availability

Data sharing not applicable to this article as no data- sets were generated or analysed during the current study.

## Abbreviations

CNPase: 2′,3′-Cyclic nucleotide 3′-phosphodiesterase
MIA: Maternal immune activation
MS: Maternal separation
Poly-I:C: Polyinosinic-polycytidylic acid
